# Evaluating Leucine, Isoleucine, and Valine Ratios in Mixed Cortical Cell Cultures Following Cortical Trauma: An In Vitro Assessment

**DOI:** 10.3390/ijtm5030042

**Published:** 2025-09-10

**Authors:** Ezek Mathew, Nathan Jones, Katherine Hernandez, Sterling B. Ortega, Rob Dickerman

**Affiliations:** Department of Microbiology and Immunology, The University of North Texas Health Science Center, 3500 Camp Bowie Blvd, Fort Worth, TX 76107, USA

**Keywords:** branched-chain amino acids, BCAA, mixed cortical culture, brain injury, traumatic brain injury

## Abstract

**Background/Objectives:**

Traumatic brain injury (TBI) remains the most common cause of morbidity and mortality in adolescents and adults. Although numerous animal and human studies have demonstrated the beneficial effects of branched-chain amino acids (BCAA) treatment on various models of brain injury, the optimal concentration and mechanism of action have not been elucidated.

**Methods:**

Based on our prior work, we hypothesized that a 2:1:1 ratio of BCAAs promotes neuronal regrowth and repair. Using in vitro mixed cortical cultures (composed of CNS cells, including neuronal and glial cells), we recapitulated the mechanical damage induced by TBI using the scratch assay model. We evaluated various concentrations of BCAA to promote the regrowth of CNS cells after mechanical damage.

**Results:**

A 2:1:1 ratio of leucine: isoleucine: valine was observed to yield superior regrowth rates at the 48 h time point across various concentrations when compared to a 1:1:1 ratio and even a 4:1:1 ratio. In addition, both 2:1:1 and 4:1:1 ratios offered multiple instances of accelerated regrowth, where less than 5% of the wound remained unhealed.

**Conclusions:**

The importance of leucine ratios in the context of BCAA treatment for TBI was demonstrated by the superior CNS cell regrowth offered by the 2:1:1 ratio.

## 1. Introduction

Traumatic brain injury (TBI) is a leading cause of death and disability across the human lifespan, with survivors often experiencing permanent neurological and cognitive impairments [1]. Worldwide, it is estimated that 69 million people sustain a TBI each year, with very young children, young adults, and elderly populations being among the most susceptible [2–4]. For years, there have been numerous attempts at treating TBI with a variety of medications, vitamins, and fatty acids. Recent studies have shown the beneficial effects of branched-chain amino acids (BCAAs) as preventative and neurorestorative [5–7]. A growing body of preclinical and clinical evidence supports BCAA supplementation as a promising therapeutic approach following TBI [8–12]. The three essential BCAAs—leucine, isoleucine, and valine—cannot be synthesized endogenously and must be obtained through diet. While the roles of isoleucine and valine are less clear, leucine has long been recognized for its role in brain metabolism and repair [13–15]. Leucine is the most abundant, versatile, and critical of the three BCAAs [7]. Leucine is also responsible for up to 50% of the nitrogen delivered to the brain, underscoring its importance in normal physiological functions ranging from protein synthesis to modulating biochemical reactions. In addition, leucine modulates neurotransmitter production, particularly glutamate, which is directly relevant to conditions characterized by excitotoxicity [7,16,17].

One of the primary functions of leucine is the de novo synthesis of glutamate [7]. Under physiological conditions, astrocytes remove glutamate from the synaptic cleft, functioning to prevent excessive stimulation and excitotoxicity. These glia then amidate the glutamate to glutamine by the enzyme glutamine synthetase, consuming ammonia in the process. Therefore, this mechanism serves to detoxify glutamate and ammonia while generating glutamine, which is not toxic to the neuron [18]. This biochemical process is known as the glutamate:glutamine cycle [18,19]. After traumatic brain injury, excess glutamate is released in a rapid fashion; this excess thus overwhelms the physiological capacity of the glutamate:glutamine cycle [19–21]. In TBI, this initial excess release of excitotoxic glutamate leads to a series of overwhelming ionic and metabolic events that, if not halted, will eventually lead to cell death [18]. Importantly, when leucine, together with isoleucine and valine, is administered after TBI, these BCAAs help regulate glutamate metabolism and sustain synaptic neurotransmitter pools [19–21]. Yudkoff et al. first postulated a leucine–glutamate shuttle that occurs after TBI, which is a substrate-dependent bidirectional reaction that assists in attenuating the extremely high levels of glutamate after injury [19].

Interestingly, several TBI studies have examined leucine supplementation alone, with consistently negative outcomes [19,22–24]. These findings suggest that leucine is not optimally effective when administered in isolation. Instead, leucine should be delivered in combination with isoleucine and valine, to maintain the temporal balance of amino acid homeostasis [8,19]. To date, numerous TBI studies have administered all three BCAAs together with varying ratios. Matsumoto et al. looked at BCAA ratios and found that a 2:1:1.2 (leucine:isoleucine:valine) ratio was effective in the context of brain-damaged mice [24]. Cole et al. looked at BCAA’s ability to restore cognitive function in mice and found that a BCAA ratio of 1:1:1 was adequate [9]. In a clinical study, Aquilani et al. observed that a slightly valine-enriched ratio of 2.5:1:3 produced a significant functional recovery in patients weeks after severe TBI [11,25]. Beyond TBI, Elango et al. looked at feeding habits and growth in neonatal pigs and reported that the optimum BCAA ratio was 1.8:1:1.2 (leucine:isoleucine:valine) for their use case [26]. Consistent with these findings, our prior work using a 2:1:1 ratio demonstrated robust preservation of both cognitive and motor function in mice following severe TBI, along with a significant reduction in neuropathological markers [8].

In order to bridge findings from in vivo models to a controlled and mechanistic in vitro setting, we employed mixed cortical cultures (MCCs)—primary cultures derived from neonatal murine cortices that, upon maturity, contain neurons, astrocytes, oligodendrocytes, and microglia in physiologically relevant proportions [27–31]. This heterogeneous composition is critical for modeling post-TBI glutamate dysregulation, as astrocytes mediate glutamate clearance, neurons contribute to excitatory signaling, and microglia and oligodendrocytes influence both inflammation and repair. MCC plates offer the advantage of maintaining these interactions while providing a reproducible platform for high-content injury modeling, such as the scratch assay, which simulates the focal mechanical damage observed in TBI.

Given the variability in reported ratios and the potential for synergistic effects among BCAAs, further investigation into the precise balance of leucine, isoleucine, and valine is warranted, particularly in the context of neurorestoration after TBI. To address this gap, we utilized an in vitro MCC scratch assay to model TBI-induced mechanical injury and directly compared the effects of 2:1:1, 4:1:1, and 1:1:1 leucine:isoleucine:valine ratios on CNS cellular recovery

## 2. Materials and Methods

### 2.1. BCAA Concentration Preparation

All experiments were approved on 5 March 2022 by the University of North Texas Health Science Center Institutional Animal Care and Use Committee (IACUC 2021–0045).

All BCAA constituents (leucine, isoleucine, valine) were purchased from Sigma-Aldrich (St. Louis, MO, USA). This leucine plasma peak concentration of 343 μM (micromolar) was reported in our previous in vivo study using a murine model under the following conditions. In our prior study, mice received a single oral gavage of a BCAA mixture (leucine 335 mg/kg, isoleucine 168 mg/kg, valine 168 mg/kg), equating to a 2:1:1 ratio at a concentration of 20 mL/kg. Plasma leucine levels were measured via liquid chromatography–mass spectrometry at multiple time points, with the measured peak concentration occurring at 45 min post-gavage, establishing a realistic range for BCAA concentrations [8]. Concentrations in this present study thus included 1 μM, 10 μM, 30 μM, 100 μM, 300 μM, and 1000 μM of BCAA at a 2:1:1 ratio of leucine:isoleucine:valine by weight of each constituent. At the aforementioned concentrations, both a 4:1:1 ratio and a 1:1:1 ratio of leucine:isoleucine:valine by weight were also investigated. The concentrations were mixed in sterile deionized (DI) water, which served as a vehicle control. When mixed into cell culture, the media was cDMEM (composed of Dulbecco’s modified Eagle’s medium (DMEM, Fisher Scientific, Waltham, USA) + 10% fetal bovine serum (FBS, Gemini Bio, West Sacramento, CA, USA) + 1% sodium pyruvate (Fisher Scientific, Waltham, MA, USA) + primocin (Fisher Scientific, Waltham, USA). Thus, this cDMEM media served as another control.

### 2.2. Mixed Cortical Culture

Primary CNS cultures were derived from post-natal murine cortices as previously described and briefly mentioned here [31,32]. The brains of mouse pups (at post-partum days 0–2) were harvested. After the euthanasia of the pups, the cranial vault was opened, and the brain was mechanically removed. The cortex was isolated, and care was taken to ensure the hippocampus, brainstem, and cerebellum were not extracted. After extraction, the meninges were removed, along with the olfactory bulbs. The cortex was dissociated using a scalpel, digested with trypsin (Fisher Scientific, Waltham, USA), and incubated. After 15 min, cDMEM was added to stop the digestion process, after which cells were mixed, spun down, and washed. After another spin and straining through a 70-micron cell strainer, the cells were properly dissociated. The extracted mixed cortical culture (MCC) hosts cell populations of neurons, astrocytes, oligodendrocytes, and microglia. The MCC cells were plated at a concentration of 0.5 × 10^6^ cells/mL in 24-well plates. Cells were incubated at 37 °C, with 5% CO_2_; after 3 days of growth, the media was exchanged. After 10 more days of growth, confluence was achieved in all wells. At maturation, the MCC plates included neurons, astrocytes, oligodendrocytes, and microglia [27–31].

### 2.3. Plating of Cells in 24 Well Plates and Scratch Assay

The scratch assay is a well-established protocol that allows for the examination of recovery after mechanical damage [33,34]. In the context of brain injury, this procedure is meant to recapitulate aspects of mechanical damage induced by TBI on cortical tissue [33,34]. Wells were washed three times in cDMEM before the scratch process was initiated. Thereafter, all of the old media was drained from each well, and 950 μL of cDMEM was added to each well, one well at a time. Within a negative pressure cell culture hood„ the scratch assay was performed according to sterile protocol. A vertical scratch was drawn from the top of the well down to the bottom, centered at the midline, as depicted in Figure S1. 12 replicates were performed, with three wells on one plate, across four plates. To perform the scratch, a new sterile 200 μL pipette tip was used for each well that was scratched, to prevent cross-contamination. Temperature was maintained by storing all plates in the incubator when not undergoing procedures. One investigator (E.M.) performed the scratches to maintain consistency. Due to concern that subsequent washing could strip away scratched cells, there was no washing after the scratch was performed. In instances where vertical scratches were incomplete due to the pipette tip skipping over the well surface, or in instances where scratches were deviated, those wells were considered abnormal and excluded. Thus, one 10 μM well of 1:1:1 ratio was excluded and replaced with an additional well at the same concentration. The same procedure occurred for one 100 μM well of 2:1:1 ratio, and for a single 1 μM well of 1:1:1 ratio. Thereafter, 50 μL of treatment modality was applied and mixed into 950 μL of media in each well. Treatments included the vehicle control, which consisted of 50 μL of DI water in 950 μL of media, or media control, which consisted of 50 μL of cDMEM, amounting to 1 mL of total cDMEM in each well. For other treatment conditions, 1 μM, 10 μM, 30 μM, 100 μM, 300 μM, and 1000 μM of protein mixture was added to each well, for 1:1:1, 2:1:1, and 4:1:1 concentrations. All treatment groups had 12 wells, allowing for comparisons.

### 2.4. Photomicrographs and Scratch Area Quantification

**Figure 1.**
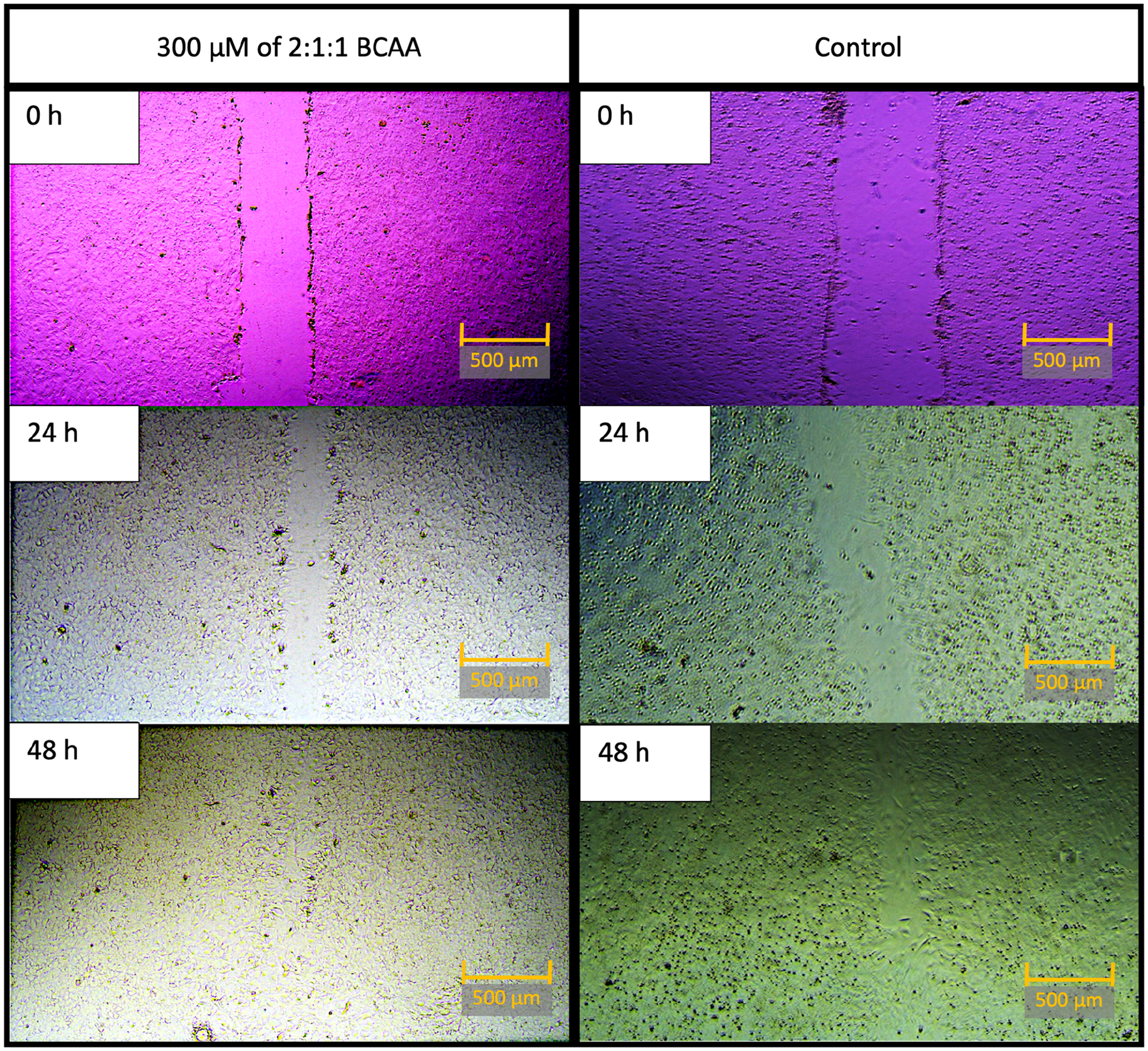
Representative well after treatment with 300 μM of 2:1:1 BCAA, on the left; control well images are on the right. All images were taken at 0 h, 24 h, and 48 h time points. Note: For the sake of visual clarity, all images were post-processed with sharpness (+100%). To optimize visibility, pictures on the left were optimized with contrast (+80%), and pictures on the right were optimized with contrast (+40%). Scale bar equals 500 μM.

After the scratch procedure, all wells were imaged to eliminate any abnormal wells or wells with cell clumping presenting as a three-dimensional mass instead of a two-dimensional monolayer. For each well, two different sites were imaged, 1/3 of the distance from the top of the well and 2/3 of the distance from the top of the well. Care was taken to ensure that the scratch was midline, and magnification was set to 4× optical zoom. The same image settings were kept for subsequent images. Images were taken immediately after treatment; these served as the baseline (0 h) images. 24 h and 48 h after the baseline image, the wells were imaged again at the previous two locations. An example well after treatment with 300 μM of 2:1:1 BCAA is shown in Figure 1. As cell proliferation across the scratch was often irregular, an area measurement was generated using an established scratch wound healing tool, named “Wound Healing Size Tool” [34]. This software calculated the number of pixels that are uncovered by cells. In the context of a scratch assay, this represents the “open wound”. As biological regrowth rates are variable, especially due to the differing cellular subcomponents of the culture, the average value of wound area was taken from both images for each well. To account for possible heterogeneity due to the deflection of the pipette tip during mechanical scratching, the following equation, Equation 1, was used to quantify the percentage of open wound, when compared to the original scratch area. All wells were normalized based on the count of unhealed pixels measured at the 0 h time point, immediately after the scratch procedure.


(1) Percentage of Open Wound=(Total Unhealed Pixels at×hours/Total Unhealed Pixels at0h)×100

### 2.5. Statistical Analysis

All experimental measurements were obtained from independent wells, which received one treatment condition per well. As two images were recorded per well or each time point, the open wound percentage between each time frame was averaged. Thereafter, across each time frame, comparisons of the open wound percentage were made within each ratio. First, one-way analysis of variance (ANOVA) was used to make a comparison between the 1:1:1 vehicle control and the 1:1:1 concentrations (1 μM, 10 μM, 30 μM, 100 μM, 300 μM, and 1000 μM). In the same manner, another one-way ANOVA was used to make separate comparisons between the 1:1:1 media control and the 1:1:1 concentrations. This process was repeated for the other ratios. For comparisons that reached a significant overall F-test (α = 0.05), we proceeded to make post hoc comparisons between the single control and each concentration using pairwise *t*-tests with Holm’s correction. This allowed us to consider the familywise error rate and control for multiple comparisons. Statistical significance was defined as *p* < 0.05 after correction.

### 2.6. Measurement of Accelerated Healing

While the metric of open wound percentage area allowed us to aggregate information on wound closure over time, there were instances where the scratch appeared almost completely closed during visual inspection. We thought it was important to note the test conditions this occurred, as there was almost complete healing in some cases. Thus, we set an arbitrary cutoff value and sought to identify wells where less than 5% of the scratch remained unhealed, translating to greater than 95% healing after treatment. By selecting a stringent cutoff, we aimed to highlight cases where the healing process appeared to be notably faster than expected. This approach allowed for a more direct comparison of healing rates across our experimental conditions.

## 3. Results

Analysis of the scratch closure at 24 h and 48 h was performed using light microscopy, along with the automated wound healing tool for quantification. The primary metric was the measurement of the open wound percentage area at these time points when compared to the 0 h (baseline time point). With this metric, lower percentages of open wound areas indicated increased healing after the mechanical insult. No statistically significant differences were found at the 0 h time point between controls and concentrations, for any ratio. Additionally, no statistically significant differences were found between vehicle control and media control for any concentration or for any time frame.

### 3.1. Wound Healing for the 2:1:1 Ratio of Leucine:Isoleucine:Valine

**Figure 2.**
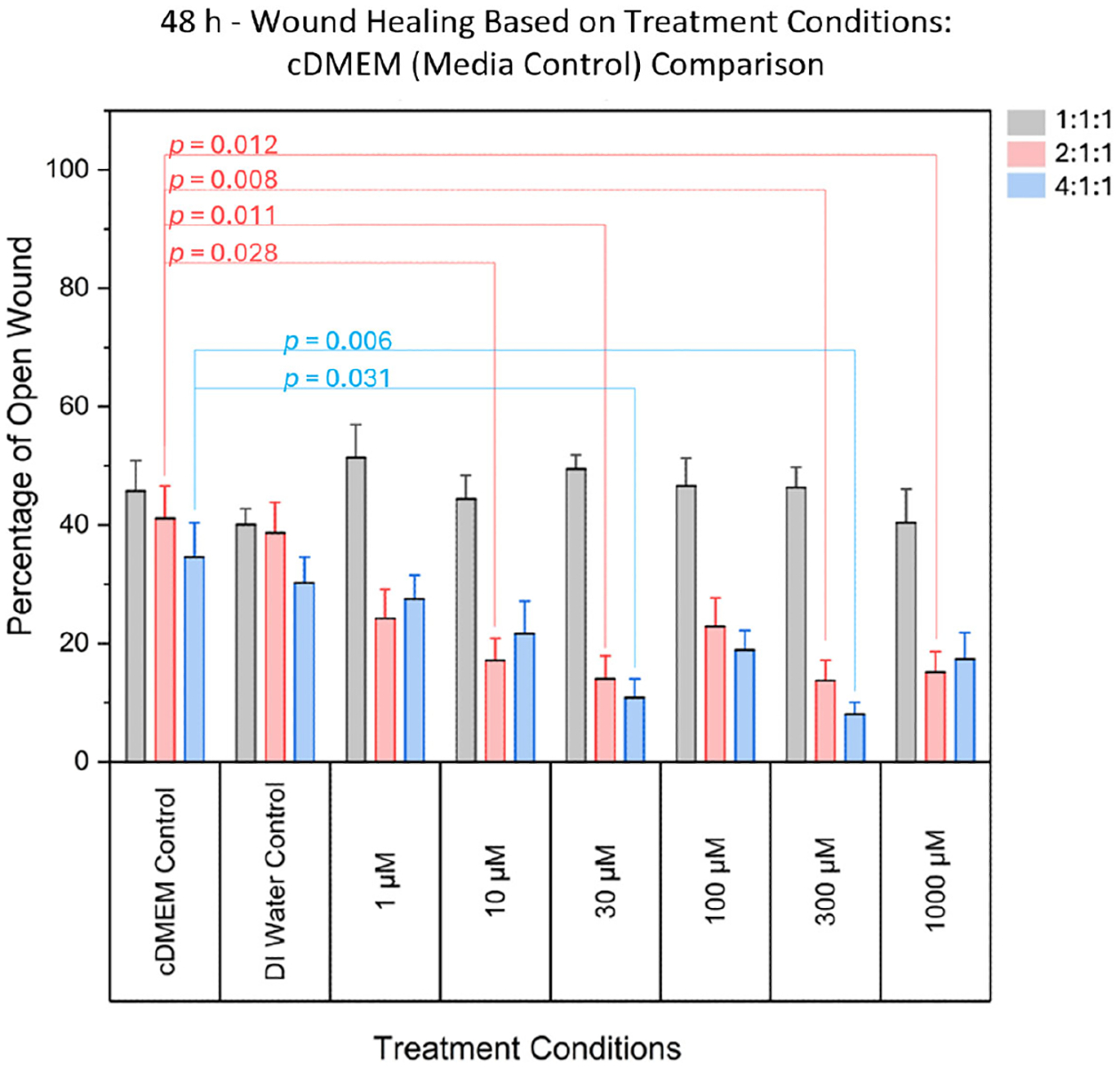
Comparison of the percentage area of open wound (representing the percentage of damaged area) between DMEM (media control) and various concentrations. Significant differences were found for various treatment concentrations, for the 2:1:1 ratio, and for the 4:1:1 ratio. Significant differences were not found in 1:1:1 ratio treatments when compared to controls. Grey bars denote the 1:1:1 ratio, pink bars denote the 2:1:1 ratio, and blue bars denote the 4:1:1 ratio.

At 48 h post-scratch, significant differences were found between the normalized open wound percentage when comparing cDMEM media controls to treatment concentrations for the 2:1:1 ratio (F(6, 77) = [5.045], *p* = 0.00021). More specifically, these differences were most notable at intermediate and high concentrations (10 μM, 30 μM, 300 μM, and 1000 μM), suggesting a concentration-dependent enhancement in wound closure, as summarized in Figure 2. No significant effects were observed at the lowest (1 μM) or moderate (100 μM) concentrations. Detailed statistical methods, including ANOVA and post hoc analyses, are provided in the Materials and Methods section. Individual *p*-values for the associated concentrations are summarized in Table 1.

**Figure 3.**
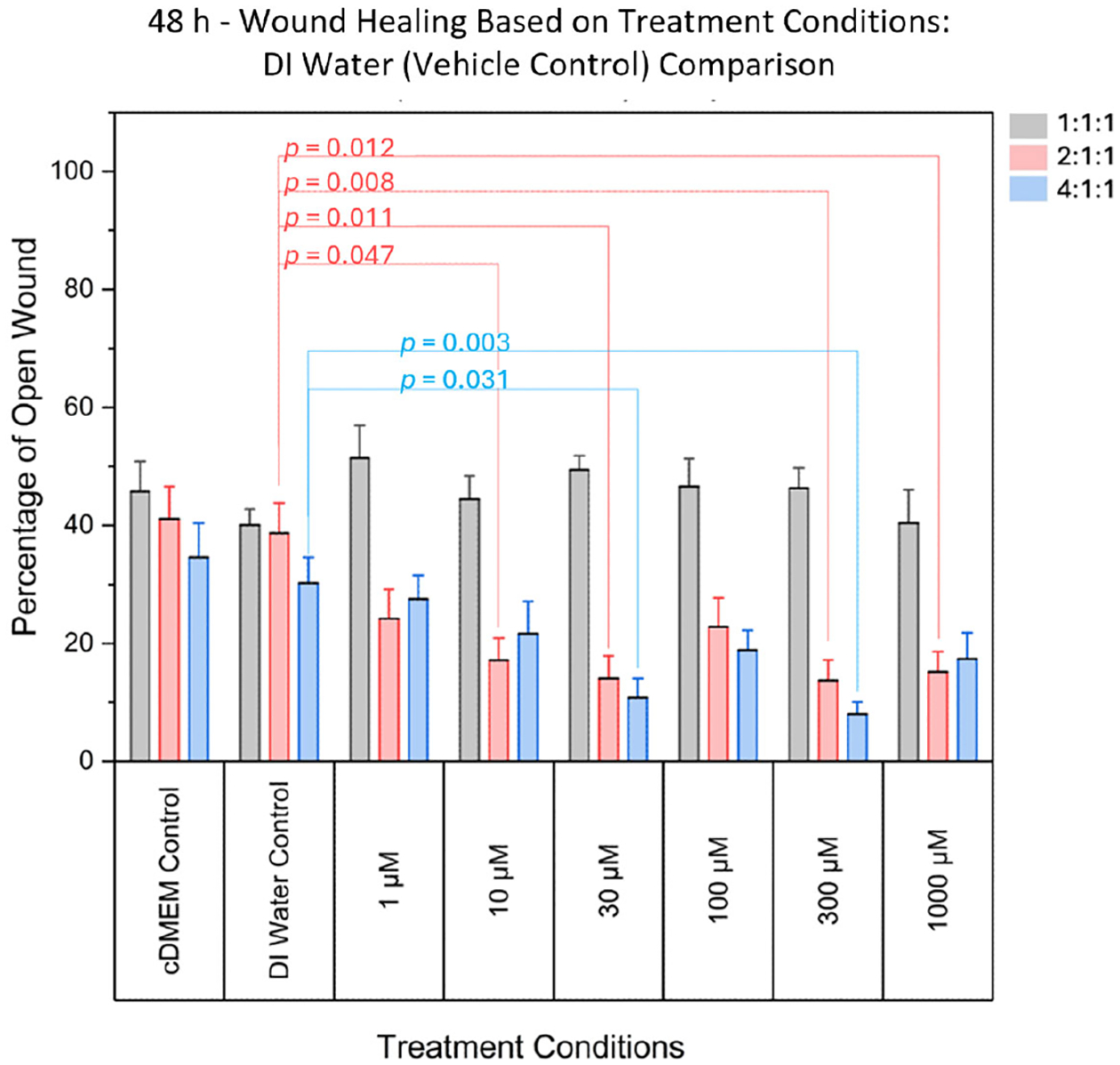
Comparison of the percentage area of open wound (representing the percentage of damaged area) between DI Water (vehicle control) and various concentrations. Significant differences were found for various treatment concentrations, for the 2:1:1 ratio, and for the 4:1:1 ratio. Significant differences were not found in 1:1:1 ratio treatments when compared to controls. Grey bars denote the 1:1:1 ratio, pink bars denote the 2:1:1 ratio, and blue bars denote the 4:1:1 ratio.

At 48 h post-scratch, one-way ANOVA was used to assess whether there was a statistically significant difference in open wound percentage between vehicle control (DI water) and treatment concentrations. Significant differences were observed in normalized open wound percentage when comparing vehicle controls (DI water) to treatment concentrations for the 4:1:1 BCAA ratio (F(6, 77) = 4.365, *p* = 0.00076). These effects were most prominent at 10 μM, 30 μM, 300 μM, and 1000 μM, indicating enhanced wound closure at intermediate and higher concentrations, as shown in Figure 3. No significant effects were detected at the lowest (1 μM) or moderate (100 μM) concentrations. Individual *p*-values are presented in Table 1, and the data is summarized in Figure 3.

No significant differences were found at the 24 h time point. Overall, the vast majority of concentrations demonstrated statistical significance, despite the heterogeneity intrinsic to mixed cortical culture.

### 3.2. Wound Healing for the 4:1:1 Ratio of Leucine:Isoleucine:Valine

At 48 h post-scratch, analysis of the 4:1:1 BCAA ratio revealed statistically significant differences in normalized open wound percentage when comparing cDMEM media controls to treatment concentrations (F(6, 77) = 5.091, *p* = 0.00019). The differences were most evident at 30 μM and 300 μM, where enhanced wound closure was observed relative to control conditions. No significant effects were detected at 1 μM, 10 μM, 100 μM, or 1000 μM, suggesting that only specific intermediate concentrations provided measurable benefit in promoting repair. These results are summarized in Figure 2, and individual *p*-values are presented in Table 1.

In a separate comparison using vehicle controls (DI water), one-way ANOVA again indicated a significant overall difference in wound closure across concentrations for the 4:1:1 ratio (F(6, 77) = 4.523, *p* = 0.00056). Post hoc analyses identified 30 μM and 300 μM as the only concentrations that significantly reduced wound area compared to vehicle controls. No significant effects were observed at 1 μM, 10 μM, 100 μM, or 1000 μM. These findings further support the conclusion that the 4:1:1 formulation exhibits concentration-specific efficacy rather than a broad concentration-response effect. The corresponding data are visualized in Figure 3 and summarized in Table 1.

At 24 h post-scratch, no significant differences were found. In summary, for the 4:1:1 ratio, two concentrations reached significance in yielding reductions in open wound percentage.

### 3.3. Wound Healing for the 1:1:1 Ratio of Leucine:Isoleucine:Valine

**Figure 4.**
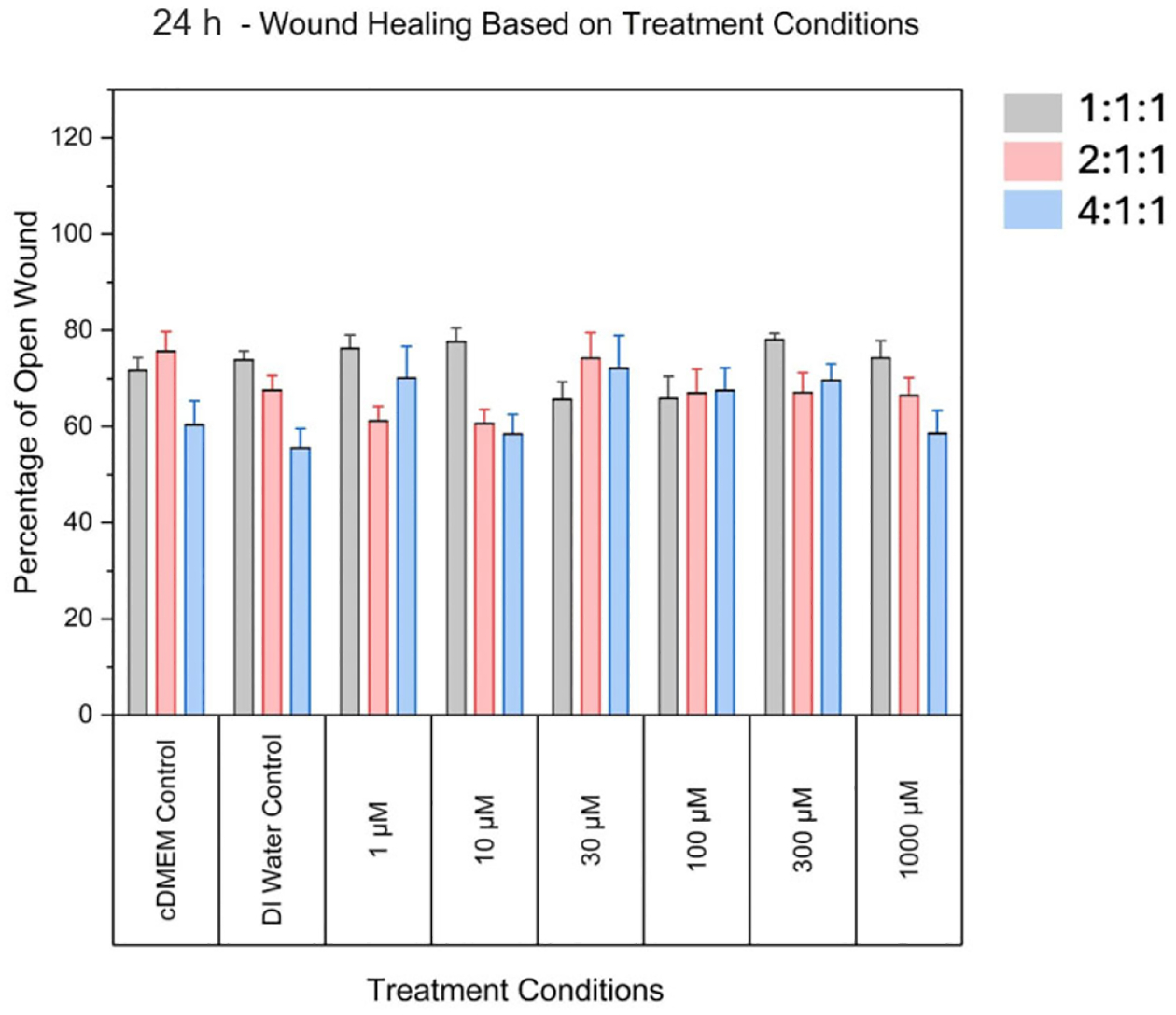
Comparison of the percentage area of open wound (representing the percentage of damaged area) between controls and various ratios at the 24 h time point. No statistically significant differences were observed at this time point for any groups, when compared to both controls. Grey bars denote the 1:1:1 ratio, pink bars denote the 2:1:1 ratio, and blue bars denote the 4:1:1 ratio.

When comparing the effect of the 1:1:1 ratio to the media and vehicle controls, there were no significant differences found at 48 h post-scratch (Figures 2 and 3). Additionally, at 24 h after the scratch, significant differences were not found between the controls and 1:1:1 treatment concentrations (Figure 4), which was the same result as for the 2:1:1 concentrations and 4:1:1 concentrations for this time frame.

### 3.4. Differential Wound Closure Response to BCAA Ratios

**Figure 5.**
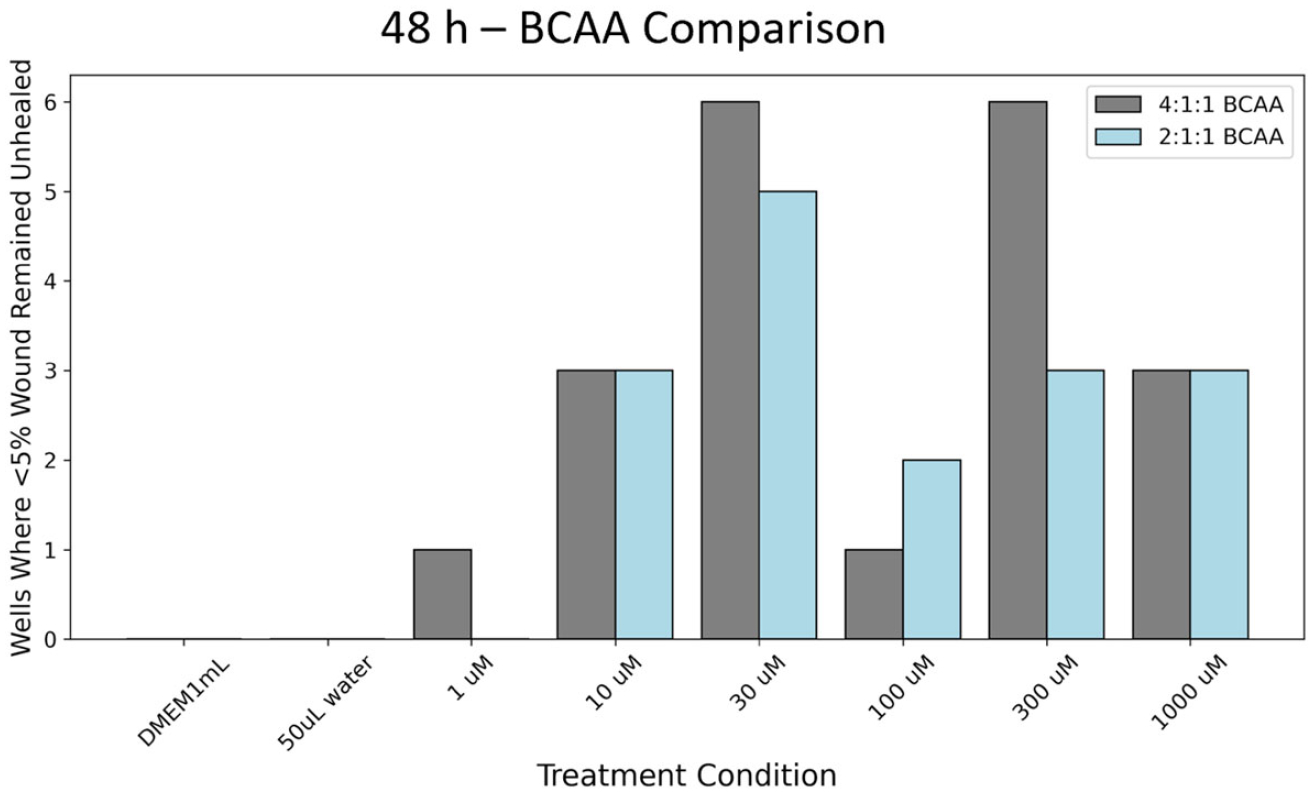
Count of wells where less than 5% of the wound remained open and unfilled with cells. Note: 12 wells total were tested for all conditions. Grey bars denote the 4:1:1 ratio and blue bars denote the 2:1:1 ratio.

While the data notes significant differences in wound closure, it is difficult to visualize the rate of closure. After collecting closure percentages for individual wells, this data was compiled in Figure 5. For the 2:1:1 BCAA treatments at the 48 h time point, there appeared to be multiple instances where healing progressed rapidly for all tested BCAA concentrations. With the same conditions, for the 4:1:1 BCAA treatments, 10 μM, 30 μM, 100 μM, 300 μM, and 1000 μM concentrations demonstrated accelerated healing, where less than 5% of the scratch remained unhealed. This phenomenon was not seen for either the controls or the wells treated with a 1:1:1 concentration. For the 1:1:1 concentration, at no time point was there less than 5% of the scratch area unhealed.

### 3.5. Overall Results

Overall, comparing the amount of open wound (% unhealed) in the 1:1:1 ratio to the 2:1:1 ratio or 4:1:1 ratio yielded reproducible results. The 1:1:1 ratio yielded results that were similar to those of controls across all tested concentrations. The 4:1:1 BCAA ratio treatment yielded significant differences in the damaged area, indicating a less open wound at the 30 μM, and 300 μM concentrations. However, the 2:1:1 BCAA ratio treatment demonstrated even better wound healing across more tested concentrations. For the 2:1:1 ratio, significant differences were observed in the damaged area at the 10 μM, 30 μM, 300 μM, and 1000 μM.

**Figure 6.**
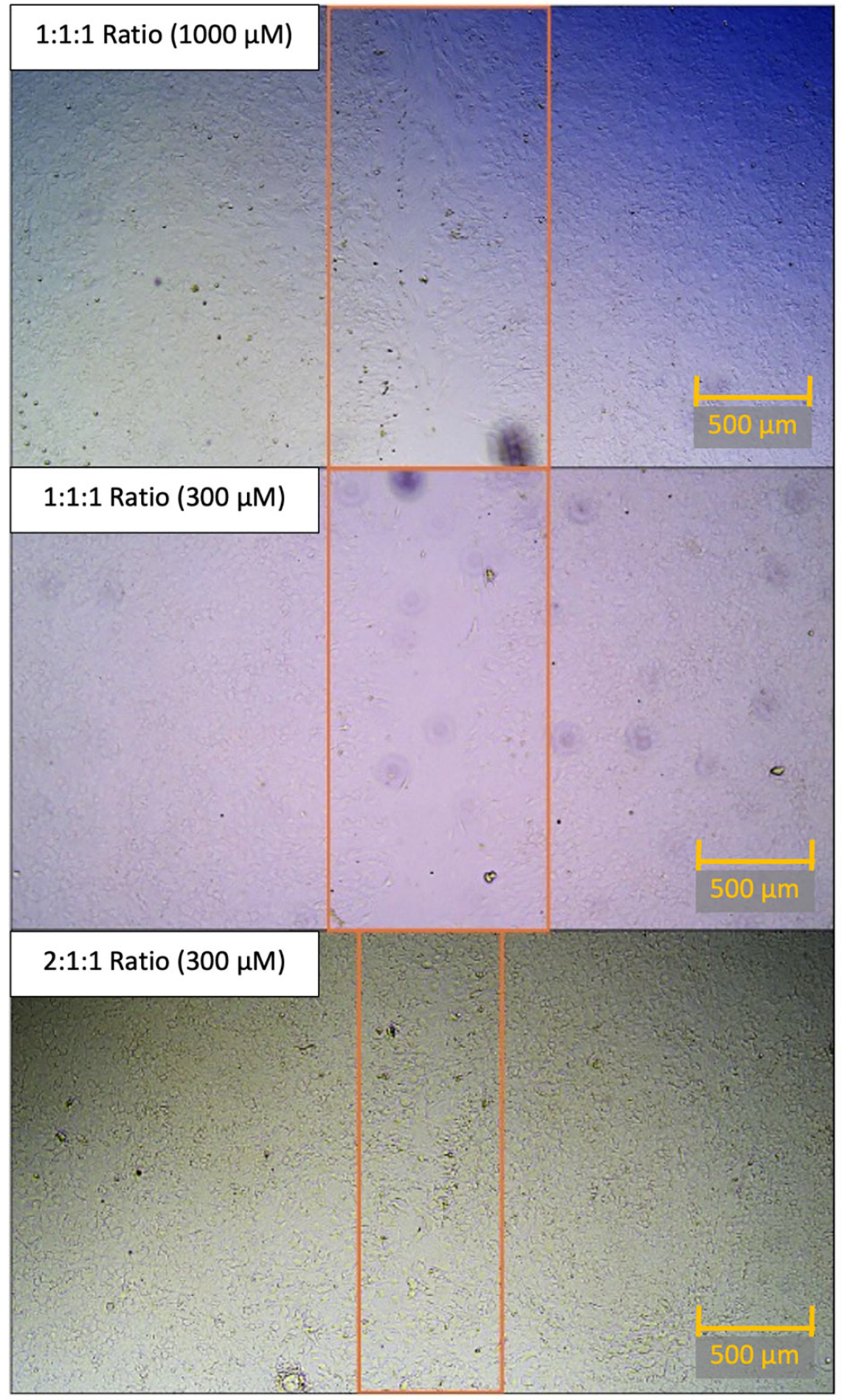
Comparison of scratch closure in top-performing wells of each BCAA ratio at 48 h. Head-to-head comparison of the best-healing concentration of 1:1:1 concentration (top panel), best-healing concentration of 1:1:1 concentration (at 300 μM), and best-healing concentration of 2:1:1 concentration (300 μM) at 48 h post-scratch. This best-healing concentration of 1:1:1 ratio (1000 μM) had an 11.51% open wound area averaged between two images when compared to the 0 h open wound area. This best-healing concentration of 1:1:1 ratio (300 μM) had a 24.51% open wound area averaged between two images when compared to the 0 h open wound area. This best-healing concentration of 2:1:1 ratio (300 μM) had 0.40% open wound area, averaged between two images, when compared to the 0 h open wound area. Note: For the sake of visual clarity, all images were post-processed with sharpness (+100%) and contrast (+65%). Scale bar (orange) equals 500 μM. The orange rectangle is used to demonstrate the bounds of the open wound area.

While we performed statistical testing on the average values across 12 wells for each concentration, for each ratio, the difference in closure is difficult to visualize. Thus, for a more granular comparison, we compared the best-healed wells of the 1:1:1 concentration to the best-healed wells of the 2:1:1 concentration. When comparing the photomicrography of the best-healed well in the 1:1:1 concentration (1000 μM) or 1:1:1 concentration (300 μM) to one of the best-performing wells of the 2:1:1 BCAA concentration (300 μM), the difference in cell repair across the scratch becomes more apparent (Figure 6).

## 4. Discussion

Since the first studies on BCAA use in the context of traumatic brain injury, there have been numerous studies demonstrating beneficial findings with varying ratios of leucine, isoleucine, and valine [19,22–26,35–39]. Further elaboration of the beneficial effect across various ratios is discussed herein, along with consideration for possible mechanisms of action. Cole and colleagues utilized a 1:1:1 ratio of leucine:isoleucine:valine and demonstrated that BCAA treatment restored cognitive function in an injured hippocampus. Interestingly, this restorative effect occurred without affecting the concentration of BCAAs in the contralateral non-injured hippocampus. Moreover, BCAA-treated shams did not have elevated central nervous system levels of BCAA, suggesting that injured regions of the brain have the capacity to pull BCAA from the systemic circulation when necessary [9,10]. This theory of injured brain tissue pulling systemic BCAA, based on required demand, is further supported by the findings that BCAA levels are very low in patients with severe TBI, immediately after injury [10,40]. Aquilani et al. performed two impressive studies utilizing an intravenous BCAA infusion with a ratio of 2.5:1:3 (leucine:isoleucine:valine) in severe TBI comatose patients and found improvement in nearly 70% of the BCAA-treated group [11,25]. Lastly, Matsumoto and Elango utilized very close ratios of 1.8:1:1.2 (leucine:isoleucine:valine) and a 2:1:1.2 ratio of the same constituents in their respective studies; both groups found these ratios to be effective [24,26]. Building upon this prior knowledge, our group recently published regarding the neuroprotective and neurorestorative effects of a 2:1:1 ratio of leucine:isoleucine:valine in severely brain-damaged mice [8]. We based the use of a 2:1:1 ratio on the overwhelming demand for leucine after TBI, as well as the natural distribution of BCAA in animals is generally accepted as 2:1:1 [11,25,41]. However, further study was needed to optimize the ratio of leucine for the treatment of TBI.

The difficulty in treating TBI arises due to the heterogeneous presentation of patients, who display high variability in the severity of injury and type of injury. Rotational, impact, penetrating, blast, or several combined mechanisms of injury, along with eloquence of the area injured, may yield differences in recovery [8,11,25]. Considering the clinical reality, we elected to proceed with a published in vitro mixed cortical cell culture model [28,31,32,42–44]. The scratch assay was used to mirror mechanical trauma induced to the brain parenchyma by TBI and is a well-established protocol used in prior literature [33,34]. The effects of BCAA treatment on wound closure in the context of monoculture would not be sufficient, as the interplay between neuronal and glial elements would be lost. Thus, we utilized a mixed cortical culture cell model, which hosts populations of neurons along with glial cells. It must be noted that MCC is also prone to inconsistent heterogeneity due to the varying amounts of neurons, astrocytes, oligodendrocytes, and microglia. These subpopulation differences are reflective of the variance in cell populations seen in human brain tissue. When compared to culturing homogenous populations of cells, this in vitro MCC model is more reflective of clinical reality [28,42–44]. Conversely, these cell population differences could greatly influence the rate of scratch repair, as seen with the variance of the open wound area, even with control treatments. As an example of a physiological correlate, varying levels of brain tissue subpopulations, such as microglia, could modify recovery after TBI. When considering the phenotypic effects of the proinflammatory M1 or anti-inflammatory M2 microglia in the context of TBI, variance in recovery may increase further [45–48]. These differences could also increase the variability of response to BCAA treatment, but more research is needed for further elucidation of this interplay.

In addition to the heterogeneity of the MCC itself, we acknowledge several important limitations that warrant future investigation. Following mechanical injury, the relative abundance of glial cells, such as astrocytes and microglia, may increase through reactive gliosis, potentially influencing healing outcomes independently of neuronal repair. Although our study did not quantify shifts in cell composition post-scratch, this would be a valuable future direction. Future studies will incorporate immunocytochemical labeling, such as NeuN for neurons, GFAP for astrocytes, Iba1 for microglia, and Olig2 for oligodendrocytes. This will allow for better assessment of cortical culture population dynamics and allow for better characterization of cell-type-specific responses to BCAA treatment. Additionally, while no overt cytotoxic effects were observed under microscopy at any BCAA concentration, formal viability testing was not conducted. To address this, we plan to incorporate cell viability assays during future experimentation to confirm that BCAA treatment does not impair cellular survival, especially at higher concentrations. While the MCC model provides a controlled environment to evaluate cellular repair processes, it cannot capture complex neurological functions such as sensorimotor recovery, learning, or memory. These functional outcomes are hallmarks of meaningful therapeutic efficacy in human TBI. Therefore, the present study should be viewed as a mechanistic exploration of BCAA-mediated effects on early cellular repair. Ongoing and future in vivo studies are needed to determine whether these cellular benefits translate into functional improvements in behavior, cognition, and long-term neurological outcome.

While this in vitro model allows for examination of repair dynamics, we acknowledge that this model does not encompass the full pathological complexity of human TBI, particularly the contributions of neuroinflammation and white matter degeneration. These features are critical in moderate to severe TBI and represent important therapeutic targets in clinical populations. Although we did not directly quantify inflammatory cytokines or immune signaling cascades, the mixed cortical culture (MCC) system employed here includes neurons, astrocytes, oligodendrocytes, and microglia, which are key cellular mediators of injury response and inflammation [49]. Thus, while molecular markers of inflammation were not assessed in this study, the cellular substrates capable of initiating inflammatory responses were present. While this may influence certain inflammatory and repair dynamics, our model was selected a priori to dissect specific biochemical responses to BCAA treatment in a tightly controlled system.

While murine models have limitations in replicating the full complexity of human white matter, they do possess well-characterized myelinated tracts, such as the corpus callosum, internal capsule, and anterior commissure [50,51]. These models remain widely used in TBI research, supported by established injury paradigms and extensive preclinical data. In our study, we utilized a mixed cortical culture (MCC) model derived from postnatal mouse cortex, which preserves key central nervous system cell types, including oligodendrocytes, which are the myelin-producing cells of the CNS. Necrotic cell death in myelinated brain regions is a hallmark of both acute and secondary injury cascades following TBI, contributing to structural degradation, blood–brain barrier disruption, and long-term neurocognitive deficits [52,53]. Although we did not directly test for necrotizing white matter damage in this study, we have cell constituents that are responsible for white matter necrotic damage present in the MCC. Our MCC model was intentionally designed to isolate early cellular responses to BCAA treatment, particularly focusing on reparative dynamics. While it does not model the full complexity of moderate-to-severe human TBI, it offers a tractable and reproducible system for mechanistically probing BCAA ratio effects on wound closure and recovery. This controlled platform lays the groundwork for subsequent translational studies in more complex in vivo systems.

The intrinsic heterogeneity of the MCC required large numbers of samples to achieve statistical significance, underscoring the biological heterogeneity that mirrors clinical TBI. Despite this complexity, treatment with the 2:1:1 BCAA ratio consistently enhanced wound closure across multiple concentrations, as quantified by the open wound area after scratch injury was induced. While the 100 μM concentration almost achieved significance (*p* = 0.06), the aforementioned variability could be an explanation for not meeting the significance cutoff. Even with a mixed in vitro culture representative of the heterogeneity of regions affected during TBI, multiple concentrations of 2:1:1 BCAA were seen to accelerate recovery after mechanically induced damage. Additionally, two concentrations with the 4:1:1 ratio did appear to yield a statistically reduced open wound area when compared to controls. While a neurorestorative effect may be present, the rate of this repair might not be on par with the 2:1:1 BCAA ratio. For all concentrations, a lack of significance in wound area was seen with the 1:1:1 ratio of leucine, isoleucine, and valine, further demonstrating the importance of BCAA ratios. The 1:1:1 ratio may have eventually led to a healing process over time, but it was not observable within the evaluated time points. The lack of observable benefit with the 1:1:1 ratio further underscores the importance of optimizing the relative composition of BCAA constituents. These findings support the hypothesis that elevated leucine levels, when balanced appropriately with isoleucine and valine, play a key role in facilitating cellular repair mechanisms following injury.

One theory behind the finding that the 1:1:1 ratio had no significant difference in the rate of scratch area closure as compared to the DMEM or water is that the rapidly increasing demand for leucine after TBI requires a higher ratio of leucine to isoleucine and valine. Previous studies have mentioned the competition for transporters among the BCAA, specifically leucine and valine [40]. This paradigm is supported in our results, as we kept the leucine dosages the same across all ratios and only reduced the concentration of isoleucine and valine to attain a 1:1:1 ratio by weight. In theory, the decreased amounts of isoleucine and valine could reduce the competition for receptors/transporters, which could allow leucine to combat glutamate via the leucine-glutamate shuttle [10,28,40]. Conversely, the 4:1:1 ratio may provide an excessive amount of leucine, resembling the ratios used in studies that reported negative outcomes with leucine alone [19,22–24]. This could explain the reduced performance of the 4:1:1 ratio in comparison to the 2:1:1 ratio, although the results were still largely beneficial.

It is interesting to note that no significant differences were found in the wound area between treatments and controls for the 24 h time period. While it is difficult to evaluate, perhaps the neurotherapeutic effect of BCAAs is a chronic phenomenon that takes multiple days to yield an effect. While this is difficult to test in vitro with these conditions, long-term studies evaluating the impact of BCAA treatment on TBI models could be a fruitful future direction. While the mechanical damage due to TBI could be an acute phenomenon, the long-term recovery after injury is of extreme clinical significance. If BCAA treatment does truly increase in effectiveness over longer periods of time, this knowledge would be highly impactful in guiding patient therapy. While in humans the rate of TBI recovery is highly variable, we have shown that with appropriate BCAA dosages and ratios, increased demand for leucine by neural tissue can be satisfied and the rate of recovery can be accelerated at later time frames beyond 24 h.

As with all treatments, toxicity is a cause for concern, especially in sensitive tissues such as the brain. We considered this and sought to include an excessively high concentration of BCAA for all ratios. Our prior study examined the in vivo bioavailability of BCAA in the mouse model and measured the leucine concentration plasma peak to be 343 μM [8]. To examine the potentially toxic effects of BCAA, a supraphysiological concentration of 1000 μM was also included among the treatment conditions. However, this concentration was also found to significantly enhance wound repair in the MCC scratch assay for the 2:1:1 ratio and the 4:1:1 ratio. While no overt toxicity was observed under microscopy, further viability assays are necessary to confirm safety at higher concentrations. The observation that a beneficial effect is seen even in extremely high dosages demonstrates the potential superiority of BCAA treatment for TBI when compared to traditional pharmacotherapies.

## 5. Conclusions

BCAA treatment at the 2:1:1 ratio significantly enhanced wound closure in a mixed cortical culture model, emphasizing the therapeutic relevance of optimized leucine dosing in TBI. Compared to other ratios, 2:1:1 showed the most consistent benefit, likely reflecting the increased metabolic demand for leucine following injury. While the results of this study align with conclusions drawn from animal models and clinical literature, this is the first in vitro assessment of BCAA repair capacity in the context of cortical culture. Future studies will be undertaken to further elucidate the roles of the glial constituents of the repair mechanism while seeking to understand the neuro-immunological mechanisms that may accelerate treatment after TBI.

## Supplementary Material

Supplementary Material

**Supplementary Materials:** The following supporting information can be downloaded at https://www.mdpi.com/article/10.3390/ijtm5030042/s1. Figure S1: Demonstration of the scratch wound healing being applied in the context of cortical cell culture.

## Figures and Tables

**Table 1.** Summary of *p*-values for BCAA ratio treatments compared to controls across concentrations at 48 h post-scratch, after Holm’s correction.

Concentration (μM)	2:1:1 vs. Media *p*-Value	2:1:1 vs. Vehicle *p*-Value	4:1:1 vs. Media *p*-Value	4:1:1 vs. Vehicle *p*-Value
1	0.519	0.874	1.000	1.000
10	**0.028**	**0.047**	0.919	1.000
30	**0.011**	**0.018**	**0.031**	**0.031**
100	0.357	0.605	0.412	0.746
300	**0.008**	**0.012**	**0.006**	**0.003**
1000	**0.012**	**0.019**	0.412	0.746

Significant values are indicated in bold text.

## Data Availability

The original contributions presented in this study are included in the article/Supplementary Materials. Further inquiries can be directed to the corresponding author.

## Abbreviations

The following abbreviations are used in this manuscript:

TBI: Traumatic brain injury
BCAA: Branched-chain amino acids
μM: Micromolar
DI: Deionized
DMEM: Dulbecco’s modified eagle’s medium
cDMEM: Complete dulbecco’s modified eagle’s medium
FBS: Fetal bovine serum
MCC: Mixed cortical culture
ANOVA: Analysis of Variance
