# Supplementary material for: Evaluating Leucine, Isoleucine, and Valine Ratios in Mixed Cortical Cell Cultures Following Cortical Trauma: An In Vitro Assessment
Source: Int J Transl Med (Basel). Author manuscript; Available in PMC 2025 Dec 4. (PMC12674610; doi:10.3390/ijtm5030042)
Supplement: Supplementary Material [file NIHMS2118258-supplement-Supplementary_Material.pdf]

Evaluating Leucine, Isoleucine, and Valine Ratios in Mixed Cortical Cell Cultures Following Cortical Trauma: An In Vitro Assessment

Ezek Mathew, Nathan Jones, Katherine Hernandez, Sterling B. Ortega and Rob Dickerman *

Department of Microbiology and Immunology, The University of North Texas Health Science Center, 3500 Camp Bowie Blvd, Fort Worth, TX 76107, USA; ezekmathew@my.unthsc.edu (E.M.); nathanjones5@my.unthsc.edu (N.J.); katherinehernandez@my.unthsc.edu (K.H.); sterling.ortega@unthsc.edu (S.B.O.)
* Correspondence: rob.dickerman@unthsc.edu

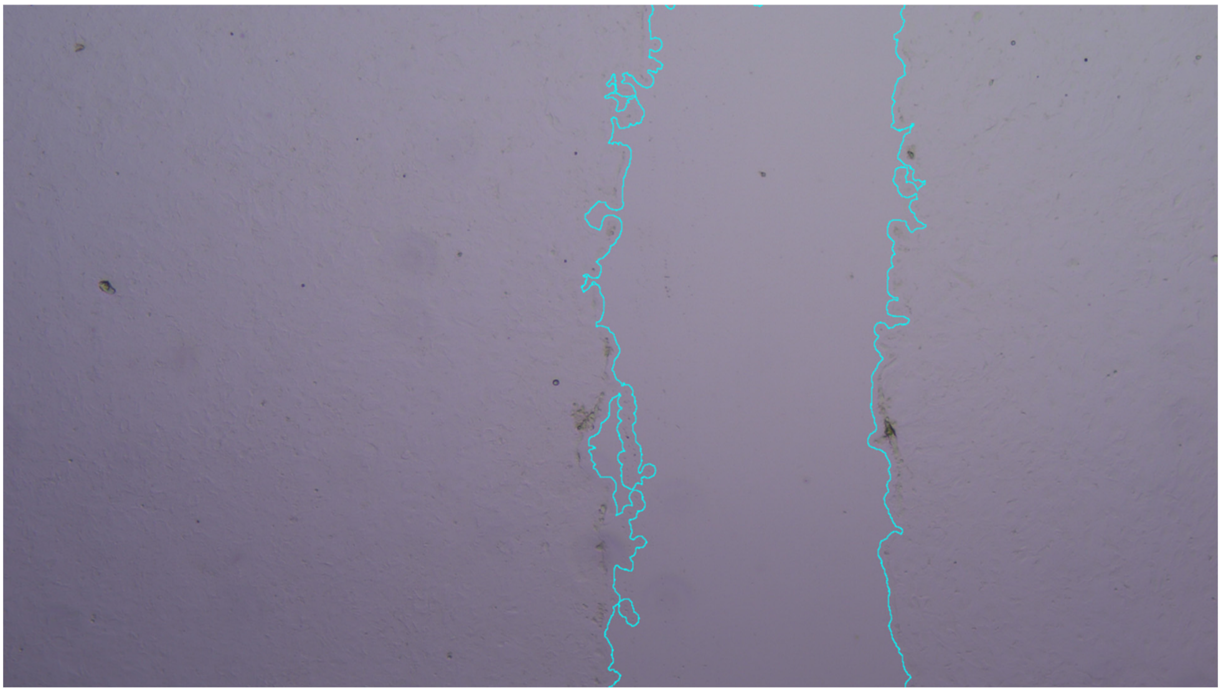

Supplementary Figure S1: Demonstration of the scratch wound healing being applied in the context of cortical cell culture.
